# MicroRNA and ROS Crosstalk in Cardiac and Pulmonary Diseases

**DOI:** 10.3390/ijms21124370

**Published:** 2020-06-19

**Authors:** Montserrat Climent, Giacomo Viggiani, Ya-Wen Chen, Gerald Coulis, Alessandra Castaldi

**Affiliations:** 1 Humanitas Clinical and Research Center—IRCCS, Via Manzoni 56, 20089 Rozzano, MI, Italy; montserrat.climentsalarich@humanitasresearch.it; 2 Department of Biomedical Sciences, Humanitas University, 20090 Pieve Emanuele, MI, Italy; giacomo.viggiani@gmail.com; 3 Hastings Center for Pulmonary Research and Division of Pulmonary, Critical Care and Sleep Medicine, Department of Medicine, Keck School of Medicine, University of Southern California, Los Angeles, CA 90033, USA; yawenc@usc.edu; 4 Department of Stem Cell Biology and Regenerative Medicine, Keck School of Medicine, University of Southern California, Los Angeles, CA 90033, USA; 5 Department of Physiology and Biophysics, and Institute for Immunology, University of California Irvine, Irvine, CA 92697, USA; gcoulis@uci.edu

**Keywords:** microRNAs, oxidative stress, ROS, cardiac disease, pulmonary disease

## Abstract

Reactive oxygen species (ROS) affect many cellular functions and the proper redox balance between ROS and antioxidants contributes substantially to the physiological welfare of the cell. During pathological conditions, an altered redox equilibrium leads to increased production of ROS that in turn may cause oxidative damage. MicroRNAs (miRNAs) regulate gene expression at the post-transcriptional level contributing to all major cellular processes, including oxidative stress and cell death. Several miRNAs are expressed in response to ROS to mediate oxidative stress. Conversely, oxidative stress may lead to the upregulation of miRNAs that control mechanisms to buffer the damage induced by ROS. This review focuses on the complex crosstalk between miRNAs and ROS in diseases of the cardiac (i.e., cardiac hypertrophy, heart failure, myocardial infarction, ischemia/reperfusion injury, diabetic cardiomyopathy) and pulmonary (i.e., idiopathic pulmonary fibrosis, acute lung injury/acute respiratory distress syndrome, asthma, chronic obstructive pulmonary disease, lung cancer) compartments. Of note, miR-34a, miR-144, miR-421, miR-129, miR-181c, miR-16, miR-31, miR-155, miR-21, and miR-1/206 were found to play a role during oxidative stress in both heart and lung pathologies. This review comprehensively summarizes current knowledge in the field.

## 1. Introduction

Pathologies of the cardiac and pulmonary systems in their broad spectrum of etiology, including among others heart failure, cardiomyopathies, lung cancer, and acute lung injury, rank among the leading causes of mortality and have a significant impact on health and healthcare of patients. Tremendous efforts and advancements in understanding critical cellular and molecular processes during the development and pathogenesis of these conditions have established an increased contribution of oxidative stress. Over the past few years, numerous studies have reported concomitant expression signature of reactive oxygen species (ROS) and small noncoding microRNAs (miRNAs), which are key players in many pathophysiological processes [1,2]. This recent focus set the premise to study the crosstalk between redox signaling and disease-specific miRNAs in the cardiac and pulmonary systems.

ROS are among the primary oxidants endogenously produced by eukaryotic cells, mostly as by-products in processes that include aerobic metabolism and cellular respiration, and host response to infection and injury; they also seem to be pivotal in both intra- and intercellular redox signaling pathways. The major source of ROS originates from mitochondrial production [3], mainly due to complexes I and III function in the respiratory chain [4,5,6]. However, other non-mitochondrial enzymes and protein complexes also produce ROS: among these are nicotinamide adenine dinucleotide phosphate oxidase (NOX) and nitric oxide synthases (NOSs) [7], xanthine oxidase [8,9], the α-ketoglutarate dehydrogenase complex [10,11], including dihydrolipoamide dehydrogenase [12,13,14,15], and d-amino acid oxidases [16,17,18]. Nitric oxide (NO), for instance, produced from sources such as endothelial NOS, contributes to vascular homeostasis, whereas under conditions of oxidative stress such as inflammation, NO interaction with other ROS potentiates cellular damage. Furthermore, these highly reactive particles can be exogenous as well, as they can be freely found in the gaseous environment. Stable ROS, such as H_2_O_2_, due to their non-radicality and the capacity to migrate across membranes, are cardinal to redox biology by activating signaling pathways to initiate biological processes. For instance, essential transcription factors such as activator protein-1 (AP-1), nuclear factor kappa B (NF-κB), and/or nuclear factor erythroid 2 like 2 (Nrf2) have been shown to be affected by the redox state with subsequent nuclear translocation and binding to DNA [19,20,21,22,23,24,25,26]. During homeostasis, ROS are kept in check by antioxidant molecules, which can either neutralize their oxidation potential or inhibit the steps that generate them.

MiRNAs are 20–22-nucleotide long noncoding RNA molecules that act as post-transcriptional regulators of gene expression. Since their initial discovery in *Caenorhabditis elegans* [27], thousands of miRNAs have been discovered [28] and their function investigated. However, the ‘miRBase’ database represents just the tip of the iceberg and most of the miRNA repertoire remains unexplored. MiRNAs are transcribed by RNA polymerase II into primary miRNAs (pri-miRNAs), then cleaved within the nucleus to hairpin-structured precursor miRNAs (pre-miRNAs) by a protein complex including Drosha and DGCR8, and finally moved to the cytoplasm by Exportin 5 (a Ran-GTP dependent transporter) where they are further cleaved by Dicer to unstable double-stranded miRNA duplexes. The duplex is further processed into a single-stranded guide RNA and becomes part of the RNA-induced silencing complex (RISC) together with Argonaute proteins. Indeed, two mature miRNAs can be generated from the same pre-miRNA precursor, referred as -5p and -3p, respectively originated from the 5′ or 3′ arm of the precursor. The RISC complex recognizes and silences target genes based on the coupling of the miRNA with the 3′ untranslated region (UTR) of the gene mRNA. The partial sequence complementarity between miRNA and target is sufficient to achieve gene silencing, with most of the ‘seed’ sequence of the miRNA (from nucleotide 2 to nucleotides 7 or 8) pairing to the 3′ UTR of the mRNA. Post-transcriptional gene silencing is achieved through either the inhibition of translation, or the degradation of the target mRNA, resulting in both cases in protein downregulation.

Due to broad target recognition, miRNAs are involved in all cellular processes including redox balance and ROS production. MiRNAs that are regulated by oxidative stress originated from several sources, such as UV, hydrogen peroxide, ionizing radiation, and antitumoral agents [29], have been termed ROSmirs and regulate the expression of their targets in response to ROS [30,31]. The activation of inflammatory cells under pathological conditions and release of cytokines induce ROS production, which can ultimately cause DNA damage and lead to cell death. Cellular events resulting in oxidative stress, including the downregulation of antioxidants, have been associated directly or indirectly with miRNA expression changes in diseases of both heart and lung compartments.

**Figure 1:**
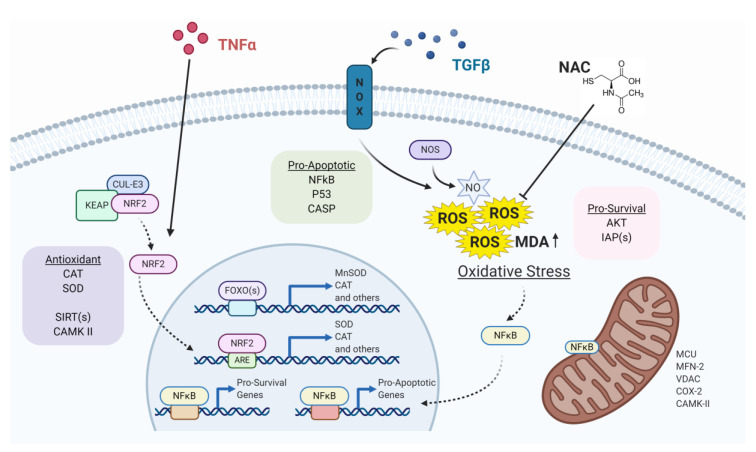
Selected key players in oxidative stress for which crosstalk with miRNAs in cardiac and pulmonary diseases is discussed in the present review.

With this review, we aim to highlight the most recent and relevant literature on the crosstalk between miRNAs and ROS in several cardiac and pulmonary pathologies. We will discuss studies describing either mechanistic correlation between miRNAs and oxidative stress-related genes or modulation of miRNA expression by ROS. Thus, studies in which ROS production have only been used as a read-out of oxidative stress following miRNA expression changes will not be included. In Figure 1, we show selected key players of oxidative stress that interact with miRNAs in cardiac and pulmonary diseases, providing a summary of the molecules mentioned in the present review.

## 2. MiRNAs and Oxidative Stress in Cardiac Diseases

### 2.1. Cardiac Hypertrophy

Cardiac hypertrophy consists of the enlargement of the heart due to the increase in size of cardiomyocytes in response to any stress that causes the heart muscle to work more; the causes can be physiological, i.e., hypertrophic response to exercise, or pathological, e.g., with long-standing hypertension, valvular disease, or any condition perpetuating pressure or volume overload. Extracellular stimuli, including angiotensin II (AngII), endothelin-1 (ET-1), isoproterenol (ISO), phenylephrine (PE), and tumor necrosis factor alpha (TNFα), can induce myocyte hypertrophy through the activation of signaling pathways downstream transduction factors, e.g., mitogen-activated protein kinases (MAPK), protein kinase C (PKC), NF-κB, calcineurin, and tyrosine kinases that can be activated by ROS [32,33]. Mitochondrial dysfunction and dysregulation can also directly impact morphological and biochemical (mal-)adaptation of cardiomyocytes [34,35]. Several miRNAs have been involved in the regulation of oxidative stress by targeting ROS generators, antioxidant pathways, and antioxidant effectors [36] in cardiac hypertrophy, having either positive or negative effects on the cardiac function [37,38]. In this section, we initially focus on miRNA gene targeting that leads to ROS generation, then on ROS regulation of miRNAs and finally miRNA regulation of mitochondrial genes involved in oxidative stress during cardiac hypertrophy.

#### 2.1.1. MiRNA Induction of Oxidative Stress in Cardiac Hypertrophy

Several miRNAs have been shown to induce oxidative stress in cardiac hypertrophy, either due to their upregulation, by targeting antioxidant genes, or their downregulation, by enabling expression of target genes.

Wang et al. reported that miR-154-5p expression increased in in vitro and in vivo models of AngII-induced cardiac hypertrophy [39]. Increased levels of miR-154-5p were sufficient to trigger cardiomyocytes hypertrophy and correlated with increased oxidative stress and inflammation. MiR-154-5p was found to directly target arylsulfatase-b (ARSB) [39], which plays a critical role in sulfate reduction in cardiomyocytes, contributing to anaerobic respiration. ARSB downregulation has been reported to inhibit mitochondrial membrane potential and oxygen consumption [40]. In line with this, targeting of ARSB by miR-154-5p led to increased ROS production and activation of the NF-κB signaling pathway, contributing, at least in part, to cardiac hypertrophy [39].

FoxO transcription factors are a subgroup of the transcriptional regulators of the Forkhead family. FoxOs regulate cellular resistance to stress by repairing DNA damage caused by ROS and by regulating the expression of antioxidant enzymes such as Manganese superoxide dismutase (MnSOD) and catalase (CAT) [41]. In AngII-induced hypertrophic cardiomyocytes, FoxO3 expression has been found to be suppressed by miR-122, which was upregulated and thus contributed to oxidative stress [42].

Hu et al. reported a correlation between miR-200c and ROS in AngII-induced hypertrophic cardiomyocytes. The authors observed an enhanced expression of miR-200c during cardiac hypertrophy and showed that its inhibition in vitro and in vivo could attenuate hypertrophy not only by targeting myosin light chain kinase (MLCK) but also by modulating oxidative stress [43]. Inhibition of miR-200c prevented ROS production, lipid peroxidation, and the increase of malondialdehyde (MDA) peroxidation, and at the same time induced increased levels of the antioxidant enzyme superoxide dismutase (SOD) activity in AngII-induced cardiomyocytes [43]. This study suggested that miR-200c might promote cardiac hypertrophy by increasing ROS production, although the mechanism and specific target(s) involved remain to be understood.

In contrast with the studies described above, in which oxidative stress was induced by overexpression of miRNAs, a similar effect was achieved by the downregulation of miR-448-3p, leading to cardiac dysfunction. NOX2 is one of the major isoforms of NOX enzymes expressed in the cardiac tissue [44]. Mir-448-3p has been found to target the neutrophil cytosolic factor 1 (*Ncf1*), which encodes for the NOX2 regulatory subunit p47^hbox^. In cardiac hypertrophy, miR-448-3p expression is downregulated, allowing for the expression of *Ncf1*, which increased ROS production. Finally, the authors reported that miR-448-3p downregulation promotes cellular oxidative stress and intracellular calcium (Ca^2+^) signaling in cardiac hypertrophy [45].

#### 2.1.2. ROS Induction of MiRNAs in Cardiac Hypertrophy

Multiple studies have extensively demonstrated that treatment with pressure-increasing drugs such as sympathomimetic ISO, as well as ET-1 and AngII, can cause oxidative stress. As mentioned before, oxidative stress is a consequence of an imbalance between the production of ROS and the ability of antioxidant systems to successfully scavenge them [32], thus aberrant levels of antioxidants can generate oxidative stress as well. Ali et al. observed an increase of free radicals and lipid peroxidation in the heart of rats in which hypertrophy was induced by ET-1 or ISO. These effects were reduced in the presence of potent antioxidants, such as N-acetylcysteine (NAC) or melatonin. Interestingly, their study revealed that several miRNAs altered in cardiac hypertrophy were involved in oxidative stress, i.e., in the generation of free radicals, SOD, and CAT activity. The expression of miR-132, miR-212, and miR-152 was enhanced in both, ET-1- and ISO-induced hypertrophy and could be reverted by the presence of antioxidants, indicating that it is regulated by ROS following ET-1 and ISO treatments [46].

#### 2.1.3. Mitochondrial Oxidative Stress and MiRNAs in Cardiac Hypertrophy

Mitochondria have a high negative membrane potential, which confers a rapid accumulation of large amounts of Ca^2+^ through a very sensitive channel named mitochondrial calcium uniporter (MCU). Mitochondria can act as sponges for Ca^2+^, which, once inside the organelle matrix, triggers signals controlling many mitochondrial features, such as morphology, energy supply, ROS production, and ultimately cell death [47]. MiR-25 has been shown to target MCU in hypertrophic cardiomyocytes resulting in decreased mitochondrial Ca^2+^ concentration with consequent decrease of oxidative stress [48], thus exerting a protective role against mitochondrial-dependent oxidative injury.

Another critical gene involved in mitochondria homeostasis is Mitofusin-2 (*Mfn-2*). Also called the hyperplasia suppressor gene, it is a crucial determinant of apoptosis in cardiomyocytes undergoing oxidative stress [49]. MiR-106a has been found to be increased in hypertrophic cardiomyocytes in vivo and in vitro, increasing ROS production by targeting *Mfn-2* [50]. The inhibition of *Mnf-2* impairs the integrity and damages the function of the mitochondria, eventually resulting in a pro-hypertrophic phenotype. Overall, Guan et al. demonstrated that miR-106a negatively contributes to cardiac hypertrophy.

Kar et al. found that PE-induced hypertrophy reduced the trans-membrane potential of mitochondria, affecting the voltage-dependent anion channel (VDAC) expression, as well as mitochondrial activity and ATP generation [34]. Interestingly, the authors demonstrated that miR-28 directly targets VDAC, being responsible for its reduction and leading to an overall mitochondrial dysfunction [34]. In summary, while miR-25 inhibits it [48], miR-106 and miR-28 promote mitochondrial-derived oxidative stress in cardiac hypertrophy [34,50].

### 2.2. Heart Failure

Heart failure (HF) is a chronic condition presenting with cardiac abnormalities at a structural and/or functional level which result in a reduction of the cardiac output and/or increased intracardiac pressure either at rest or during stress. The heart is ultimately not able to keep the peripheral blood perfusion needed to supply the whole body. HF can originate from other cardiac pathological conditions (e.g., cardiac hypertrophy, myocardial infarction), ultimately leading to death in 90% of patients. The structure and function of the heart can be affected by the macromolecular damage and disruption of the cellular redox signaling caused by ROS production. Thus, oxidative stress has a vital role during the transition from compensated heart function towards HF, due to increased levels of NOX and activation of ROS sources, as well as reduced antioxidant capacity. This directly leads to oxidative damage contributing to contractile dysfunction, myocardial hypertrophy, and fibrosis, eventually leading to congestive heart failure (CHF) [32,51,52].

#### 2.2.1. MiRNA Regulation of TNFα-Dependent Oxidative Stress in HF and CHF

Tumor necrosis factor α (TNFα), an inflammatory cytokine produced during acute inflammation, plays an important role in HF and CHF, exacerbating myocyte hypertrophy [32,53]. Of relevance, TNFα can lead to the activation of Nrf2 in different cell types [54]. Nrf2 regulates cardiac remodeling during oxidative stress by inducing transcription of its downstream genes by recognizing antioxidant response elements (ARE). TNFα also regulates proteins involved in the regulation of the cellular response to stress, e.g., high-mobility group box 1 (HMGB1) [55].

Tian et al. identified three miRNAs (miR-27a, miR-28a and miR-34a) highly expressed in the myocardium during CHF [51]. The authors observed a decrease of Nrf2 protein levels in the left ventricle (LV) of a rat model of CHF following myocardial infarction (MI), with a consequent decrease of Nrf2-dependent antioxidant enzymes (HO-1, SOD2, and CAT) as well. However, *Nrf2* mRNA levels were upregulated after damage, which could represent a compensatory mechanism to increase gene expression in response to the low levels of protein. Accordingly, the authors suggested that the three miRNAs are involved in the post-translational inhibition of Nrf2. Thus, this study indicated that miR-27a, miR-28a, and miR-34a are involved in the regulation of HF-induced oxidative stress by inhibiting Nrf2 translation [51].

#### 2.2.2. MiRNA Induction of Oxidative Stress in HF

Several miRNAs have been shown to induce oxidative stress during HF through the regulation of either pro-survival, antioxidant, or mitochondrial genes.

Cardiomyocytes treated with palmitate, naturally present in a variety of food, e.g., palm oil, and known to induce ROS production [56], exhibited increased levels of miR-195. This miRNA was shown to induce ROS production and promote apoptosis by targeting Sirtuin 1 (Sirt1), a protein that plays a key role in the maintenance of cellular homeostasis [57].

The expression of miR-132, which was shown to target FOXA1, was reduced in peripheral blood of both HF patients and HF-induced rats. In vitro studies demonstrated that miR-132 overexpression in cells treated with H_2_O_2_ reduced apoptosis and oxidative stress through increasing SOD levels and reducing MDA levels [58]. In addition, miR-132 overexpression reduced Transforming growth factor-β1 (TGF-β1) and Smad3, two important pro-fibrotic factors, suggesting an overall cardioprotective role for miR-132, which was abolished by its downregulation in HF [59].

Finally, in vivo studies on induced HF identified a group of miRNAs, i.e., miR-696, miR-532, miR-690, and miR-345-3p, as expressed specifically in mitochondria isolated from the heart and involved in oxidative stress [60].

### 2.3. Myocardial Infarction and Ischemia/Reperfusion

MI is defined as myocardial cell death due to prolonged inadequate supply of oxygen to the heart tissue, known as ischemia. Once the blood supply is insufficient, necrosis of the myocardial tissue occurs within a few hours. Timely reperfusion therapies are the most effective methods to rescue the ischemic myocardium. Nevertheless, myocardial reperfusion can initiate oxidative stress through massive ROS generation, which further promotes myocardial injury inducing apoptosis and autophagy: this process is known as ischemia/reperfusion (I/R) injury. I/R injury has been adopted as a model to study MI and its mechanisms include a ROS burst during reperfusion, as well as calcium overload [61,62], resulting in increased apoptosis [63]. Conversely, SOD expression favors the post-ischemic cardiac function recovery, indicating the critical role of ROS and oxidative balance in MI and I/R. A process known as ischemic preconditioning (IPC) takes place in myocardium under repeated short ischemic insults, representing an adaptive response by which the heart develops resistance for subsequent ischemic injuries. IPC has also been adopted in experimental settings and to study MI and I/R. Generation of ROS and NO, as well as PKC and tyrosine kinases, are known to participate in the development of IPC and to activate NF-κB, regulating gene expression in response to IPC [32,64].

#### 2.3.1. Crosstalk between miRNAs and the Sirtuin Family in MI

Sirtuins (SIRTs 1-7, in mammals) are a family of seven enzymes that is involved in metabolic regulation and relies on the availability of Nicotinamide adenine dinucleotide (NAD^+^) in the cells. SIRTs are involved in oxidative stress, often acting as antioxidants [65].

Sirt1, which is involved in many biological processes, e.g., inflammation, cell death, and metabolism [66], functions by deacetylating proteins, e.g., peroxisome proliferator-activated receptor gamma (PPAR-γ) and coactivator 1 alpha (PGC-1α). PGC-1α is strongly expressed in the heart [66] and plays a key role in the regulation of mitochondrial biogenesis. MiR-22, which plays an important role in myocardial infarction and, later on, in cardiac remodeling, has been shown to participate in the damage caused by mitochondrial oxidative stress in I/R injured-cardiomyocytes [61]. Consistently, miR-22 was found to be upregulated in rats subjected to I/R and its inhibition to be sufficient to reduce the injury [67]. Du et al. elucidated the mechanism by which increased levels of miR-22 worsened the in vitro I/R-induced mitochondrial damage, demonstrating that miR-22 directly targeted Sirt1 and PGC-1α, ultimately inducing oxidative stress [67].

Clinical trials have shown that the transplantation of endothelial progenitor cells (EPC) can be used to treat acute MI, and a substantial loss of EPCs is known to depend on oxidative stress during infarction [68,69]. Wu et al. showed that, during H_2_O_2_-induced oxidative stress, expression levels of miR-126 were reduced in EPCs, which exhibited disrupted biological functions. Overexpression of miR-126 could revert the injury in EPCs by reducing oxidative stress as demonstrated by decreased ROS and MDA production and increased SOD, angiopoietin (Ang) 1, and Ang2 expression. Although the authors did not investigate the specific gene targeting through which miR-126 exerts its antioxidant function, they showed a direct activation of PI3K/Akt/SK-3b and ERK1/2 signaling pathway by miR-126, which contributed to the biological functions of EPCs [70]. Sirt1, which is known to deacetylate Akt and promote its activation, might be involved in the miR-126 regulation of this pathway.

#### 2.3.2. MiRNA Targeting of the CaMKII Signaling Pathway in MI

ROS can modulate calmodulin-dependent kinase II (CaMKII), a serine/threonine kinase, found to be increased during HF [71] and involved in I/R. Although not identified as a ‘conventional’ antioxidant, CaMKII inhibition can be beneficial against pathological oxidation [72] and a few miRNAs have been shown to regulate the CAMKII signaling pathway.

MiR-214 was identified as a critical effector molecule protecting cardiac stem cells (CSCs) from oxidative stress by targeting CaMKII [73]. Wang et al. found that exosomes originated from bone marrow-derived mesenchymal stem cells (BMSCs) could decrease apoptosis and ROS production in CSCs following oxidative stress injury. The authors demonstrated a dramatic increase of miR-214 in the BMSCs-originated exosomes that were released under hypoxic conditions, reducing in turn CaMKII expression levels [73].

MiR-145, a miRNA known to regulate cancer and vascular smooth muscle cell phenotype [74,75], has also been found to target CaMKII. It has been shown that downregulation of CaMKII by miR-145 significantly lowered intracellular calcium and suppressed H_2_O_2_-mediated calcium overload in rat ventricular cardiomyocytes [76]. MiR-145 also targeted Bcl2/adenovirus E1B 19 kDa-interacting protein 3 (Bnip3), which plays a critical function in the mitochondria, i.e., mediating apoptosis and sensing oxidative stress in the cytoplasm. Downregulation of Bnip3 by miR-145 was reported to cause a reduction in ROS production, showing a miR-145 protective role in cardiomyocytes undergoing oxidative stress as well as in the heart of mice subjected to I/R [77].

#### 2.3.3. MiRNA Fine-Tuning of the Antioxidant Program in MI

Li et al. demonstrated an antioxidative role for miR-340-5p during I/R [63]. Decreased levels of miR-340-5p were observed in in vivo I/R injury following MI, as well as in in vitro I/R in cardiomyocytes. After I/R, an increase of ROS and MDA and decrease of SOD levels were reported. However, the overexpression of miR-340-5p was able to suppress enhanced levels of ROS and MDA and also to restore the activity of SOD in I/R-induced cells. Furthermore, the authors found that miR-340-5p targeted the NF-κB pathway activator protein 1 (Act1) [63], which plays a crucial role in the intracellular signaling process leading to cell death [78], demonstrating that miR-340-5p overexpression can exert a cardioprotective role during I/R injury through negative regulation of the NF-κB signaling pathway.

MiR-23 has a controversial oxidant effect, depending on the context. In primary cardiomyocytes treated with H_2_O_2_ and in I/R injury mouse model [79], miR-23 was shown to target MnSOD, which is a critical antioxidant enzyme, located in the mitochondrial matrix where it protects cells from oxidative stress [80], suggesting that miR-23 favors ROS accumulation. In contrast, another study showed that, in acute myocardial infarction (AMI) patients, miR-23a is downregulated [81]. Of relevance, in cancer, miR-23a was found to target PTEN, a well-known phosphatase directly antagonizing PI3K signaling [82], known to be inactivated under oxidative stress [83]. In line with this, in H_2_O_2_-treated cardiomyocytes, PTEN inhibitor increased miR-23a expression, thus increasing the antioxidant activity of SOD, glutathione (GSH), and CAT, while decreasing MDA activity. Therefore, the authors suggested that the PTEN/miR-23a axis could be beneficial in AMI [81].

Nrf2-keap1 complex, which is inactive under physiological conditions, is activated under oxidative stress, dissociating and migrating into the cytoplasm, where it stimulates the expression of antioxidant response element (ARE)-containing genes [84]. Xiao et al. found that miR-24 was significantly decreased in I/R cell models of primary cardiomyocytes, and its overexpression exerted an anti-apoptotic role by targeting Keap1 [85].

MiR-133a and miR-1 have been involved in pathological processes such as cardiac hypertrophy [86,87,88]. Izarra et al. showed that only miR-133a, but not miR-1, was increased under oxidative stress conditions in cardiac progenitor cells (CPCs) [89]. The authors showed that overexpression of miR-133a could protect CPCs against cell death by targeting the pro-apoptotic genes Bim and Bmf [89]. More recently, Yu et al. studied the effects of aloe-emodin (AE), an exudate from the aloe plant, on MI injuries in vivo and oxidative insults in vitro. The authors found a protective effect of AE in neonatal rat ventricular cells (NRVCs) under H_2_O_2_ treatment mediated by the cytoprotective effects of miR-133 directly targeting caspase-3. In particular, AE treatment could invert the loss of mitochondria membrane potential induced by H_2_O_2._ in vitro, and in vivo decrease the infarct size, promoting a better cardiac function and a decrease in cardiac apoptosis and oxidative stress in the mouse heart post-MI [90].

#### 2.3.4. MiRNA and ROS Crosstalk during MI

Other miRNAs have been shown to crosstalk with MI oxidative stress-related genes. MiR-98 negatively regulates MI by inhibiting Fas/Caspase-3 signal [91]. MiR-370 was shown to have a protective role by inhibiting oxidative stress in cardiac myocytes targeting FOXO1 [92].

MiR-100 targets insulin-like growth factor-1 receptor (IGF1R), impairing the protective effects of IGF1 signaling in the setting of H_2_O_2_-induced (and thus ROS-mediated) apoptosis [93].

MiR-208 was found to target protein tyrosine phosphatase receptor type G (PTPRG) and protein tyrosine phosphatase, non-receptor type 4 (PTPN4), which are crucial regulators of ROS stimulation [94]. Moreover, miR-208 was decreased in myocardial I/R injury in vivo as well as in H_2_O_2_-treated cardiomyocytes. Thus, increased levels of PTPRG and PTPN4 caused reduced ROS production, increased expression of ROS scavengers enzymes (SOD and CAR), improved cell viability and alleviated apoptosis [95].

Lastly, miR-223-3p levels are increased in hypoxic cardiomyocytes and inhibition of miR-223-3p was able to prevent hypoxia-induced apoptosis and delay oxidative stress by inhibiting ROS generation and lipid peroxidation by targeting Kruppel like factor 15 (KLF15). Thus, decreased miR-223-3p levels also enhanced the expression of antioxidant enzymes, such as SOD, CAT, and GPx [96].

### 2.4. Diabetic Cardiomyopathy

Oxidative stress is well known to play a negative role in the development of chronic vascular complications, especially in patients with diabetes mellitus (DM) [32]. In diabetic cardiomyopathy (DCM), myocardial apoptosis is directly related to the generation of ROS in the heart, which is considered to be critical in the development and progression of DCM [97,98,99].

#### 2.4.1. MiRNA Cardioprotective and Antioxidative Role in DCM

MiR-203 expression is reduced during high-glucose (HG) treatment in myocardial cells [100]. This miRNA was found to target PtdIns-3-Kinase Subunit Alpha (PIK3CA), which, by mediating the PI3K/Akt signaling pathway, caused a reduction of oxidative stress and apoptosis in myocardial cells. In addition, in vivo experiments on diabetic mice confirmed the protective role of miR-203 on myocardial hypertrophy by targeting the PI3K/Akt pathway, which induced MDA and ROS levels in the fibrotic tissue [100]. Moreover, increased levels of miR-203 in vivo could reduce myocardial oxidative stress. Thus, in this study, the authors suggest a cardioprotective function of miR-203 in diabetic cardiomyopathy by targeting PIK3CA [100].

ROS production, induced by mitochondrial dysfunction, is a potential modulator of diabetic-induced cardiac diseases. Li et al. observed, in the heart of diabetic mice, a decrease of mitochondria cytochrome-b (mt-Cytb), a subunit of the complex III where ROS are abundantly produced. Although the authors found mt-Cytb to be a predicted target of miR-92a-2-5p, mt-Cytb was positively modulated in the presence of miR-92a-2-5p. Moreover, re-expression of miR-92a-5p in cardiomyocytes decreased ROS production and lipid deposition, ameliorating the diabetic cardiomyopathy [101].

Peroxisome proliferator-activated receptor co-activators (PGCs) play an important role in DCM [102]. Indeed, PGC-1β as well as ROS levels and cell apoptosis were increased in diabetic hearts. MiR-30c was found to directly target PGC-1β, alleviating high palmitate-induced lipotoxicity in vitro and attenuating cardiac dysfunction in diabetic mice [103].

#### 2.4.2. Cardiotoxic Role of MiRNAs in DCM

Phase II enzyme inducers, such as 1,2-dithiole-3 (4H)-thione (CPDT), are compounds that act as antioxidant enzymes through the activation of Nrf2. In vivo treatment of DCM rats with CPDT have been shown to decrease the levels of miR-503 and increase the levels of Nrf2, MDA, and HO-1. Specifically, Nrf2 has been found to be a direct target of miR-503 in myocardial cells [104], suggesting a pro-oxidative role for miR-503.

Another miRNA that is increased in the heart of diabetic mice is miR-195. Silencing miR-195 was found to reduce DCM in mice by targeting Sirt1 and Bcl-2, also attenuating cardiac hypertrophy, oxidative stress, and caspase-3 activity [105].

## 3. MiRNAs and Oxidative Stress in Pulmonary Diseases

### 3.1. Idiopathic Pulmonary Fibrosis

Idiopathic pulmonary fibrosis (IPF) is a chronic progressive disease that exhibits abnormal re-epithelialisation due to persistent injury and consequent fibrosis; patients mean survival is less than five years. Aging, genetics, and cigarette smoking have all been shown to contribute to IPF pathogenesis, but the exact underlying mechanisms have yet to be elucidated. Under physiological conditions, two main cell types constitute the alveolar epithelium: alveolar type I (AT1) cells, which play a critical role in gas exchange, and surfactant producing alveolar type II (AT2) cells, which serve as progenitors for AT1 cells. The process of transdifferentiation from AT2 to AT1 cells is crucial to ensure a successful re-epithelialisation following injury. Global changes in miRNA expression profile have been reported to happen during this process [106], suggesting a potential role for miRNAs in the regeneration of the alveolar space following injury. Several studies have shown the involvement of miRNAs [107,108,109,110,111,112,113] and oxidative stress [114] in IPF. Increased concentrations of lipid peroxidation products, oxidized proteins, and impairment of antioxidant enzyme expression have often been found in epithelial lining fluid collected from IPF patients. In vivo models that fully recapitulate the chronic features of IPF are not yet available; mouse models currently used for the study of IPF, such as injury models, e.g., bleomycin and paraquat, and genetically modified mouse models that display pulmonary fibrosis [115], develop non-chronic fibrosis that eventually resolves by itself.

#### Crosstalk between MiRNAs and ROS in IPF

The paraquat (PQ)-induced pulmonary fibrosis model is based on the toxicity of PQ, for which PQ-poisoning has been associated with high mortality rate [116]. Using the PQ-induced PF model, Liu et al. showed that ligustrazine, an antioxidant extracted from the roots and stems of *Ligusticum chuanxiong Hort (Chuan Xiong)* ameliorates lung fibrosis by inactivating the AKT/mTOR and hedgehog signaling pathway through upregulation of miR-193a, leading to increased cell autophagy and reduced fibrosis. This study demonstrated that miR-193a mediates the antioxidant protective effect of ligustrazine in pulmonary fibrosis [117].

Aging is often associated with pulmonary diseases such as IPF and it is in part regulated by the replicative senescence, i.e., irreversible cell-growth arrest due to telomere shortening. However, under DNA damage stimulus, oxidative stress can induce senescence without telomere shortening, namely premature senescence. In lung fibroblasts, the downregulation of Casein Kinase 2 Alpha 1, known as CK2, has been shown to induce both premature and replicative senescence [118]. MiR-760 and miR-186 have been found to be dramatically upregulated during replicative senescence in lung fibroblasts and to target, in synergy with miR-337-3p and miR-216b, CK2-alpha, which caused an increase of ROS generation and subsequently replicative senescence. This study elegantly described the phenomenon by which more miRNAs cooperate to downregulate the expression of the same target. The authors also reported that overexpression of these miRNAs could accelerate premature senescence in proliferating cells by targeting CK2 and therefore reduce its capability to phosphorylate p53, stabilizing p53 and at the same time increasing the levels of ROS [119].

Finally, Fierro-Fernández et al. found miR-9-5p to function as a redoximiR and fibromiR and showed that it is involved in a feedback loop with TGF- β1 [120]. TGF-β is known to interfere with redox homeostasis in IPF [121] and to enhance the expression of NOX4 in many cell types, e.g., in heart and lung [122,123,124], leading to oxidative stress and thus amplifying the fibrogenic program. The authors found miR-9-5p to be upregulated by TGF-β and to regulate in turn TGF receptor 2 (TGFR2) and NOX4, which they showed to be direct targets of miR-9-5p. Modulation of TGF-β and miR-9-5p in vitro demonstrated the existence of a feedback loop where the ROS-dependent expression of TGF-β induced miR-9-5-p expression, that in turn downregulated TGFR2 and NOX4 genes. Thus, the in vivo orotracheal instillation of miR-9-5p, before subjecting the mice to bleomycin injury, protected the lungs from fibrosis and oxidative stress. However, miR-9-5p was found dramatically upregulated in the lungs of IPF patients, as well as in the bleomycin mouse model, apparently in contrast with its protective role. The author suggested that endogenous levels of miR-9-5p were lower than the levels reached after exogenous miR-9-5p administration, accounting for its failure to protect from fibrosis [120].

### 3.2. Acute Lung Injury and Acute Respiratory Distress Syndrome

Acute respiratory distress syndrome (ARDS) is the most severe form of acute lung injury (ALI), an acute, diffused, inflammatory form of lung injury that is associated with a variety of etiologies and with a mortality of about 40%. ARDS/ALI can originate from lung infection, e.g., Gram-negative bacterial infection where endotoxins, i.e., Lipopolysaccharide (LPS), are released and cause local inflammation. Thus, ARDS/ALI is a consequence of an alveolar injury and diffused alveolar damage that impairs normal gas exchange, fluid accumulation, and clearance across the pulmonary epithelium, affecting lung compliance and increasing pulmonary arterial pressure. Cellular injury results in the release of pro-inflammatory cytokines, which attract polymorphonuclear cells such as neutrophils to the lung tissue. Their function is to release toxic and reactive macromolecules, e.g., ROS and proteases, exacerbating the damage to the capillary endothelium and alveolar epithelium. As a self-perpetuating cycle, ROS-induced oxidative stress causes upregulation of pro-inflammatory cytokines and cell adhesion molecules, further magnifying the pulmonary damage and edema [125]. Thus, the regulation of and by ROS is a key factor in ARDS pathogenesis and a better understanding of it might lead to the identification of novel therapeutic targets for ARDS. Of relevance, miRNAs have been found to play a role in ARDS and in lung inflammation, e.g., pneumonia [126].

#### Crosstalk between MiRNAs and ROS in ARDS/ALI

LPS exposure causes oxidative stress by inducing aggregation of NOX subunits, p47phox (NCF1), and gq91phox (CYBB, also known as Nox2), to form the functional NOX enzyme. NOX-derived ROS then act as inflammatory signals to activate the inflammatory response [127]. LPS regulates the p47phox subunit phosphorylation [128], as well as its expression. Under prolonged stimulation with LPS, miR-19 was found to be upregulated and in turn to negatively regulate ROS production by targeting the NOX subunit p47phox. In line with this, a miR-19 knock-out mouse model exhibited higher levels of ROS and a worsened lung phenotype compared to the wild-type. Thus, Wang et al. proposed the existence of a regulatory feedback between miR-19 and ROS induced by LPS that regulates the inflammatory response [129].

Hyperoxia-induced acute lung injury is another well-established model for ALI/ARDS [130,131]. Hyperoxia exposure is a robust source of ROS that might lead to oxidative stress. Cells exposed to hyperoxia attempt to adapt and activate repair mechanisms. However, if hyperoxia exposure is prolonged, cells undergo apoptosis, autophagy, and/or necrosis [132]. MiR-185 was shown to be upregulated in hypoxia-induced ALI due to suppression of histone deacetylase 4 (HDAC4) that is located in its promoter, and to promote DNA damage leading to hyperoxia-induced lung epithelial cell death [131]. In another study, Zhang et al. reported that a long noncoding RNA, namely FOXD3-AS1, was strongly upregulated in hyperoxia-exposed mouse lungs and human alveolar epithelial cells. They demonstrated that the FOXD3-AS1 mRNA directly bound miR-150 on several sites, acting as a sponge for this miRNA and leading to miR-150 downregulation in hyperoxia-induced models. MiR-150 is known to target p53 [133], thus its downregulation by FOXD3-AS1 is, at least in part, a cause for p53-dependent apoptosis in oxidative stress conditions. In support of their findings, the authors also showed that hyperoxia downregulated miR-150 via ROS in cells treated with H_2_O_2_ [134].

### 3.3. Airway Diseases: Asthma and Chronic Obstructive Pulmonary Disease

Asthma and chronic obstructive pulmonary disease (COPD) are among the most common airway diseases. While both display inflammation of the airways and a variable degree of obstruction, the mechanisms underlying the two diseases are wildly different.

Asthma is characterized by intermittent dyspnea, wheezing, and cough. This is due to airway hyper-responsiveness to a specific stimulus (e.g., allergen) with an excessive contraction of the airways leading to airflow limitation. However, asthma is a clinical syndrome with multiple underlying mechanisms, including abnormalities of airway smooth muscle, pathological remodeling, and faulty interplay between epithelial and mesenchymal cells. The production of ROS may be upregulated during the allergen exposure in asthma due to chronic inflammation [135]. Oxidative stress originates from a disruption of the homeostasis between high levels of ROS and antioxidant defense, which results in induction of pro-inflammatory cytokines, chemokines, and adhesion molecules. Toll-like-receptors (TLRs) and other macromolecules like transcription factors, such as NF-κB, are well-recognized players in many asthma-related inflammatory pathways [135].

COPD is a persistent respiratory syndrome due to alveolar and/or airway dysfunction with consequent impairment of the airflow after chronic exposure to noxious exogenous agents, e.g., smoke. The resulting chronic inflammation leads to permanent destruction of the respiratory parenchyma (emphysema), pathological remodeling with small airway narrowing, and mucociliary dysfunction that exacerbates mucous plugging of the airways [136]. Due to chronic exposure to toxicants, antioxidant capacity in COPD is decreased, and oxidative stress is recognized as one of the major predisposing factors in COPD pathogenesis [137]. Interestingly, some miRNAs have been reported to have a role in COPD irrespective of the smoking status of the patient, i.e., miR-122-5p and miR-218-5p/miR-15a that were found to interact with the TGF-β pathway, while the role of others was related to smoking, i.e., as Let-7 [138].

#### Crosstalk between MiRNAs and ROS in Asthma and COPD

In the search for a compound or phytochemical that could be beneficial for asthmatic patients and that could even downregulate oxidative stress in the airways, Ho et al. studied the effect of diallyl sulfide (DAS), an enriched component found in garlic, on lungs of mice treated with ovalbumin (OVA), a commonly used model of allergen-exposed response. The authors reported that, among other beneficial effects, e.g., reduced inflammation, oxidative stress was reduced in OVA-challenged mice when DAS was orally administered. The levels of Nrf2, that are diminished following OVA inhalation, were restored in the presence of DAS. The expression of several miRNAs was found to be altered in OVA-challenged mice, specifically miR-155, miR-146a, miR-146b, miR-144, and miR-34a were significantly higher while miR-34b/c lower. Following DAS administration to the OVA-challenged mice, miR-144 and miR-34a were downregulated. Both these miRNAs have been reported to downregulate Nrf2 [139,140], thus the DAS antioxidant effect is mediated, at least in part, by miR-144 and miR-34a regulation of Nrf2 [141].

Mizuno et al. analyzed RNA and miRNA expression in the lungs of 55 COPD patients and found that miR-34a and miR-199a-5p were overexpressed compared to histologically healthy lungs. In vitro studies and analysis of COPD lung tissues showed that miR-199a-5p was associated with hypoxia-inducible factor-1α (HIF-1α) expression. The authors further investigated the relationship between oxidative stress/miR-34a/miR-199a-5p in COPD and suggested that oxidative stress induces miR-34 upregulation through the upregulation of p53. MiR-34a inhibited the activation and phosphorylation of AKT, which conversely caused miR-199a-5p upregulation. Finally, miR-199a-5p reduced the expression of HIF-1α which can impair the vascular endothelial growth factor (VEGF) expression that together with AKT inactivation leads to cell apoptosis and emphysema. Although the gene expression was performed on RNA extracted from total lung tissue, the authors speculated that the described signaling cascade was activated by oxidative stress in pulmonary endothelial cells and alveolar septal cells [142]. However, one caveat of the study was that >40% of the patients analyzed had lung cancer as well, complicating the understanding of whether the signaling was specific of COPD or lung cancer.

### 3.4. Lung Cancer

Lung cancer is one of the deadliest cancers worldwide, with a 5-year survival rate of less than 18% for lung cancer patients. As for most cancers, the causes of lung cancer are a combination of environmental, genetic, and aging effects. Lung cancer can be broadly classified as small cell (SCLC, 13%) and non-small cell (NSCLC, 87%) cancers; approximately 50% of the malignancies of the lung are represented by adenocarcinomas. Lung cancer is much more common in smokers, since the inhalation of carcinogens synergizes with the generation of ROS due to chronic inflammation, leading to oxidative stress with high DNA damage potential that often leads to oncogenic driver mutations [143]. Among the fundamental dogmas for carcinogenesis is that cells and tissues acquire features that allow them to be pathological and survive: they proliferate uncontrollably, are not recognized by the immune system, adapt to their surroundings, and generate masses that have their own homeostasis. The latter point includes a perpetuated, tumor-induced inflammation, which allows continued generation of ROS and thus further DNA damage and carcinogenesis.

Of interest, lung cancer and COPD share some mechanisms of disease, such as a genetic predisposition, excessive cell plasticity, perpetuated inflammation, extracellular matrix deposition, and DNA damage, and ROS generation has been linked to the pathogenesis of both diseases. Of note, smoking is considered an etiologic factor for both illnesses.

#### 3.4.1. Crosstalk between MiRNAs and ROS to Support Lung Cancer Growth

One of the mechanisms that cancer cells use to escape the cell cycle arrest is to increase the ROS production to cause DNA damage, which is followed by proliferation and tumor outgrowth. In SCLC, overexpression of miR-17-92 cluster, that in healthy lungs induces epithelial cell proliferation during development [144,145], has been found to counterbalance the DNA damage generation induced by the inactivation of Retinoblastoma (RB) [146]. RB is a crucial regulator of entry into cell division that acts as a tumor suppressor [147], and its inactivation in SCLC causes DNA damage. MiR-20a, a component of the miR-17-92 cluster, was found to directly target cyclin E [148], a downstream transcriptional target of E2F1 that is itself upregulated following RB inactivation. Ebi et al. concluded that, following RB inactivation, miR-17-92 overexpression reduces excessive DNA damage and ROS production to tolerable levels through miR-20a targeting of cyclin E, leading to genetic instability [146] and tumor growth.

ROS have been shown to induce angiogenesis to support tumor growth by suppressing miR-199a-5p in arsenic-treated (As-T) human bronchial epithelial cells [149]. MiR-199a-5p has been involved in several carcinogenic processes, i.e., proliferation, migration, invasion, apoptosis, autophagy, and glycol-metabolism [150]. Of interest, a protective role for miR-199a-5p has been described in I/R injured cardiomyocytes, while in the lung it has been shown to play a role in development and fibrosis [151,152]. As-T cells produce elevated levels of ROS [153] that have been reported to suppress miR-199a-5p expression by increasing its promoter methylation [154]. In a more recent study, He et al. confirmed that miR-199a-5p is strongly downregulated in As-T cells and further demonstrated that ROS scavenger CAT decreased the expression of COX-2, which they found to be a miR-199a-5p target [149]. This was in line with the observed downregulation of miR-199a-5p in the presence of ROS and regulation of angiogenesis through COX-2 [155]. Furthermore, cells stably overexpressing miR-199a-5p treated with As did not overexpress COX-2 in the presence of H_2_O_2_, indicating that a ROS/miR-199a/COX-2 pathway is activated in As-T cells to induce angiogenesis [149].

#### 3.4.2. Crosstalk between MiRNAs and ROS to Suppress Lung Cancer Growth

Tumor suppressor miRNAs are usually downregulated in tumor, while onco-miRs are upregulated. However, some tumor suppressor can act as ‘sensors’ and be upregulated in cancer cells. This is the case for miR-506, a member of the X-linked miRNA cluster [156] that has been often found upregulated in NSCLC and that, at the same time, plays a pro-apoptotic/tumor suppressor function when overexpressed in cancer cells [157]. Yin et al. reported that higher expression of miR-506 correlated with increased apoptosis in lung cancer samples due to its direct targeting of the pro-inflammatory transcription factor NF-κB p65 and subsequent inhibition of NF-κB-activated anti-apoptotic genes. Specifically, the expression of miR-506 was found to be positively correlated with oxidative stress participating in oxidative stress-induced apoptosis. Yin et al. suggested that miR-506 is the link through which p53 activates pro-apoptotic protein poly-ADP-ribose polymerase (PARP) following ROS induction. Of interest, these mechanisms were only found in cancer cells but not in non-cancer cells, demonstrating the existence of a feedback loop between ROS, p53, and miR-506 that negatively regulated NF-κB p65 expression in cancer cells. In this environment, miR-506 sensed ROS and induced apoptosis, ultimately acting as a tumor suppressor [157].

#### 3.4.3. Crosstalk between MiRNAs and ROS to Induce Metastasis

MiRNAs have not only been involved in mechanisms underlying cancer initiation and progression but also in invasion and metastasis [158,159,160]. Lung cancer can often be diagnosed at late stages (III, IV) due to the absence of symptoms or the misinterpretation of them by both the patient and the physician. Thus, it is important to investigate and understand mechanisms that underlie the metastatic process in adenocarcinomas such as NSCLC, to anticipate them. Sun et al. [158] reported that downregulation of miR-99a is often associated with poor prognosis and advanced stage and metastasis in lung adenocarcinoma. Sun et al. demonstrated that, while migration and invasion of cancer cells could be decreased by transfection of a miR-99a mimic, this effect was abolished when a NOX4 construct was co-transfected [158]. In vitro and in vivo data showed that NOX4 is a target of miR-99a and its miR-99a-dependent upregulation in NSCLC accounts for the ROS production increase and the subsequent migration /invasion. Cells transfected with anti-miR-99a failed to induce migration and proliferation in the presence of the NAC, clearly demonstrating that downregulation of miR-99a favors metastasis by increasing ROS through the targeting of NOX4 [158].

#### 3.4.4. MiRNA Induction of ROS Accumulation in Lung Cancer

Li et al. found that miR-182 was overexpressed in lung adenocarcinoma and regulated ROS production [161]. MiR-182 was shown to suppress pyruvate dehydrogenase (PDH) kinase 4 (PDK4), leading to *de novo* lipogenesis of which the antioxidant nicotinamide adenine dinucleotide phosphate (NADPH) is one of the most important substrates. Thus, because of PDK4 targeting-dependent lipogenesis, miR-182 overexpression caused an increment of ROS, that further induced tumorigenesis through the JNK pathway [161]. The treatment of lung cancer cells with NAC could reduce cancer colony formation in cells overexpressing miR-182, demonstrating that miR-182 positively regulates ROS to promote tumorigenesis in lung adenocarcinoma.

#### 3.4.5. Crosstalk between MiRNAs and ROS to Favor Chemoresistance

One way for cancer cells to develop drug resistance under chemotherapy is to overexpress ROS scavengers to downregulate ROS production and thus be protected from apoptosis [162]. Wang et al. showed that the crosstalk between miR-146a and ROS is involved in the mechanism through which cancer cells resist the chemotherapeutic drug cisplatin. Downregulation of the Receptor-interacting protein 1 (RIP1) was found to increase drug sensitivity in cancer cells exposed to cisplatin by inducing apoptosis through the upregulation of miR-146a. Although the authors did not show the direct targeting of CAT by miR-146, they reported that suppression of miR-146a expression clearly restored CAT expression in cancer cells and reduced ROS accumulation and apoptosis. ROS are known to negatively regulate the inhibitor of apoptosis (IAP) family proteins [163]. RIP1 exerts a chemo-resistant role by maintaining IAP expression through the downregulation of miR-146a and consequent overexpression of CAT/reduction of ROS. Thus, a chemoresistance signaling pathway exists consisting of RIP1, miR-146a, CAT, and IAPs that can be targeted in the future to induce chemosensitization [164].

One of the proteins that has been associated with chemoresistance in lung cancer is Mucin-1 (MUC1), found to be overexpressed in NSCLC and correlated with poor survival [165]. MUC1 increased expression was found to be correlated with chemoresistance and to be ROS-dependent [166]. ROS generation observed in chemoresistant cells is in part due to the overexpression of miR-551b that suppresses CAT. Interestingly, treatment of resistant cells with a ROS scavenger caused an increase of miR-551b, suggesting a negative feedback from ROS that controls miR-551b. Finally, the authors showed that the miR-551b/CAT/ROS signaling cascade could result in MUC1 overexpression that, in turn, activated the pro-survival EGFR/AKT pathway and support chemoresistance [166].

#### 3.4.6. MiRNAs as Mediators of ‘Natural’ Antioxidant in Lung Cancer

Some nutrient components, e.g., lignans, have antioxidant capabilities. Because radiotherapy increases ROS production, there is a great interest in understanding whether and how dietary supplement can ameliorate the effects of radiotherapy by reducing oxidative stress. Flaxseed (FS), a non-toxic whole grain seed with high concentrations of omega-3 fatty acids and lignans, has been shown to play a double role when combined with radiotherapy. On the one hand, FS is potentially able to mitigate side-effects; on the other hand, it counteracts the effect of radiotherapy on the tumor [167]. Christofidou-Solomidou et al. investigated miRNA expression profile in the lung of animals under FS diet and found miR-142-3p and miR-150 to be significantly downregulated, irrespective of radiation exposure, and miR-34a to be significantly upregulated. The authors suggested that the altered expression of miRNAs may account for the antioxidant action of FS [167]. The FS induction of miR-34a overexpression is of particular interest given that a phase I clinical trial based on miR-34a mimics (NCT01829971) was performed and withdrawn in 2016 due to five immune-related adverse events. However, the miR-34 family remains a promising cancer therapeutic candidate [168]. For instance, miR-34c has been found to be downregulated in NSLC cells [169] and involved in the regulation of ROS production. The oncogene HMGB1 was identified as a miR-34c target and found indeed upregulated in NSLC. By modulating the expression of the miR-34c and/or HMGB1, Tu et al. showed that miR-34c targeting of HMGB1 led to reduced levels of ROS and apoptosis. In contrast, HMGB1 overexpression inhibited miR-34c effects on NSCLC cell proliferation, apoptosis, and ER stress [169]. Finally, the authors demonstrated that ROS levels are directly correlated to miR-34 expression in NSCLC.

Several phytochemicals have also been reported to have antioxidant properties in lung cancer. Tymoquinone (TQ), a phytochemical derived from *Nigella sativa*, has been reported to have anti-neoplastic capabilities [170,171]. Upadhyay et al. demonstrated that delivery of TQ to NCLSC could reduce the cancer growth by increasing apoptosis, and that a crosstalk between miRNAs and ROS was involved in this process. Nanoparticles, decorated with transferrin (TF-TQ-Np) to enhance their entrance into NCSLC, were used to treat A549 cells. In treated cells, the authors observed an increase of apoptosis, partly through ROS production and partly through activation of p53 signaling. P53-mediated apoptosis was found to be dependent on the overexpression of miR-34a and miR-16, demonstrated targeting the anti-apoptotic mitochondrial protein Bcl2. A positive feedback loop was found between ROS and p53 and related to the miR-34 and miR-16- dependent apoptosis following TQ-TF-nanoparticle treatment, both in vitro and in vivo. Thus, strict cooperation between miR-34a and miR-16 and ROS production exists, and it accounts for the promising anti-neoplastic effect of the TQ-TF nanoparticles [172].

Another study focused on the capability of the *Polygonatum odoratum Lectin* (POL) to induce autophagy and apoptosis in A549 [173]. Of interest, the regulation of ROS by miR-15a-3p was involved in the POL-induction of apoptosis. Specifically, the authors found that in POL-treated cells the levels of miR-15a-3p were higher and that this induced an increased phosphorylation of p53, leading to apoptosis. Treatment with NAC could reverse the POL or miR-15a-3p mimic-induced cytotoxicity, as well as the activation of p53. Although the exact mechanism by which miR-15a-3p regulates the p53 phosphorylation state was not investigated, p53 inhibitor was shown to suppress the miR-15a-3p mimic-induced apoptosis. Altogether, the data indicated that the miR-15a-3p/ROS/p53 axis is involved in POL-induced apoptosis and autophagy in A549 cells [173].

Particulate matter 2.5 (PM2.5) in polluted air has been associated with respiratory disease occurrence. Exposure to PM2.5 can induce ROS production and oxidative stress in lung cells. Li et al. have found miR-486 to be downregulated in A549 cells incubated with PM2.5 and demonstrated that the overexpression of miR-486 by a mimic suppressed the PM2.5-induced ROS generation and apoptosis. They further demonstrated that miR-486 was able to protect against apoptosis and oxidative stress by targeting PTEN and FOXO1 [174]. The correlation between miRNA regulation and antioxidant function of another phytochemical which can be found in high concentrations in white mulberry and other plants of *Moracea* family, i.e., morin, has also been investigated. Veerappan et al. were particularly interested in the protective function of morin on A549 cells exposed to PM2.5 and reported that several miRNAs (miR146a, miR21, miR222, miR24, miR421, miR210, miR101, miR93, and miR200a) were downregulated by morin. The authors also found that miR-34a, which was increased under PM2.5 exposure, got downregulated in morin pre-treated cells. Based on previous studies that showed a role for these miRNAs in the regulation of oxidative stress-related genes, the authors suggested that the antioxidant role of morin is mediated, at least in part, by miRNAs [175].

## 4. MiRNAs and Oxidative Stress in Both Cardiac and Pulmonary Diseases

**Figure 2:**
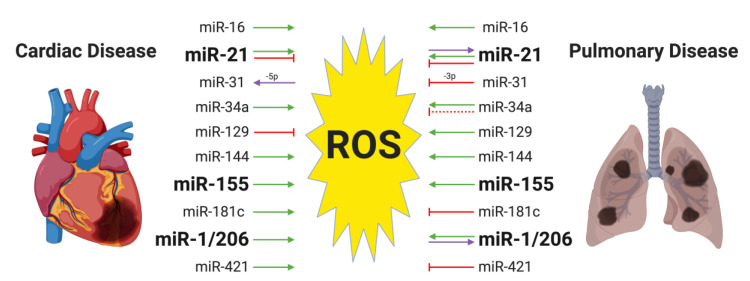
MiRNAs involved in oxidative stress in diseases of both cardiac and pulmonary systems. Green arrows, miRNA induction of ROS; red arrows, miRNA inhibition of ROS; purple arrows, ROS induction of miRNA. A dashed arrow indicates a putative contribution of miR-34a to the antioxidative function of Flaxseed. MiRNAs are listed in numerical order.

Several miRNAs have been reported to crosstalk with oxidative stress in both cardiac and pulmonary systems. In Figure 2, we summarize the findings for the ten miRNAs shared by cardiac and pulmonary settings. Three of them, miR-155, miR-21, and miR1/206, have been extensively studied in cardiac and pulmonary diseases, thus we will highlight them in the following sub-sections.

In the lung, miR-144 and miR-16 have been reported to work synergistically with miR-34a under oxidative stress conditions. As reported above, miR-144 and miR-16 cooperated with miR-34a to downregulate the antioxidant transcription factor Nrf2 [139,140,141] and the anti-apoptotic mitochondrial protein Bcl2, respectively, contributing to oxidative stress [172]. Their functions were very similar in the context of cardiac disease. In a cell line of cardiomyocytes, as well as in vivo, in the myocardium of rats subjected to I/R, miR-144 has been found to target another antioxidant transcription factor, i.e., FOXO1, preventing the myocardial I/R injury to some extent [176]. In AMI rat models and neonatal rat ventricular cardiomyocytes, miR-16 has been shown to be increased and to target beta2-adrenergic receptor (β2-AR), which is known to protect injured cardiomyocytes from hypoxia- and oxidative stress-induced apoptosis [177]. Indeed, high miR-16 levels caused decreased β2-AR expression and, consequently, induction of hypoxia and oxidative stress. Thus, both miR-144 and miR-16 were found to promote oxidative stress in cardiac and pulmonary diseases; however, there is no evidence of a potential cooperation of these two miRNAs with miR-34a in the heart. Of note, as described above, miR-34a was also reported to target Nrf2 in synergy with miR-27a and 28a in CHF [51].

Furthermore, in the lung, miR-34a has been correlated to the antioxidant function of FS in radiotherapy, and, more in general, the miR-34 family members have been suggested to be a promising therapeutic target for the treatment of lung cancer, as well as to be involved in COPD [142,167,168]. In the heart, an increase of miR-34a has been observed in pre-diabetic and diabetic patients [178], where HG levels are known to induce an accumulation of ROS. MiR-34a was found upregulated in diabetic mouse hearts and to regulate redox signaling pathways [179]. Moreover, it was reported that miR-34a upregulation in diabetic mice led to dysregulation of endothelial cells by targeting Sirt1 [180]. Interestingly, in an independent study, in vitro experiments using cardiomyocytes in HG conditions confirmed the induction of high levels of miR-34a and targeting of Sirt1 [181], ultimately leading to oxidative stress. Thus, while induction of miR-34a can exert an anti-tumoral therapeutic effect, its downregulation could be of potential benefit for the treatment of DCM.

A contrasting function between cardiac and pulmonary systems has been reported for three miRNAs, i.e., miR-421, miR-129 and miR-181c.

MiR-421 was shown to promote oxidative stress in the heart, by targeting Sirt3, which plays an antioxidative role in cardiomyocytes, and conversely to induce the gene expression of antioxidants by targeting KEAP1, the Nrf2 binding partner [182], in the lungs. Inhibition of Sirt3 expression or suppression of its activity was associated with an increase of ROS and activation of Akt signaling [71]. In the cardiac I/R settings, Liu et al. demonstrated that silencing of miR-421 in vitro decreased apoptosis, and both MDA and LDH levels, and increased SOD levels, ultimately reverting I/R effects. Sirt3, the miR-421 target, was found downregulated after I/R and its expression inversely correlated to increased expression of miR-421 [62]. In lung cancer, the regulation of ROS by miR-421 has been associated with resistance to the chemotherapy drug paclitaxel [183]. MiR-421 was reported upregulated and associated with poor prognosis in NSCLC patients [184] which was further correlated with the targeting of KEAP1. Under oxidative stress, Nrf2 is generally released from the KEAP1-Cul3 E3 ligase complex [185] and can translocate into the nucleus and activate antioxidant response elements. Knock-down of miR-421 caused levels of KEAP to rise, inhibiting Nrf2-dependent antioxidant expression, and ultimately increasing intracellular levels of ROS [183]. This study, therefore, suggests that the inhibition of miR-421, whose expression was found to be induced by beta-catenin, could increase the sensitivity of cancer cells to paclitaxel by inducing ROS-dependent apoptosis.

MiR-129-5p has been found downregulated in cardiac diseases, allowing the expression of its target HMGB1, and it was suggested that its restoration could be protective from oxidative stress. HMGB1 regulates cellular responses to stress, inflammation and tissue damage, and oxidative stress induces translocation, release, and activity of HMGB1 during inflammation and cell death [186]. HMGB1 regulation of downstream apoptosis or survival requires TNFα for its secretion and HMGB1 accumulation at sites of oxidative DNA damage can induce repair of the DNA [55]. In the serum of patients with CHF, lower levels of miR-129-5p were observed to be inversely correlated with HMGB1 levels [187]. Accordingly, when miR-129-5p was overexpressed in vivo, levels of HMBG1 were lower in the myocardial tissue. MiR-129-5p was shown to directly target HMBG1, reducing inflammatory response (TNFα, Interleukin 6) and MDA levels and increasing SOD levels in both serum of rats with CHF and cardiac cells [187]. Thus, restoration of miR-129-5p levels could play a protective role in CHF, decreasing oxidative stress, and inflammation in cardiac cells [187]. In contrast, miR-129-5p has been reported to have a pro-oxidative stress role in the lung. HDAC inhibitors, such as suberoylanilide hydroxamic acid (SAHA), have been shown to induce apoptosis in various cancer cells by increasing the production of ROS [188,189]. You et al. [190] found that miR-129-5p was upregulated by SAHA-treatment in cancer cells and, at least in part, mediated ROS increased production by targeting the antioxidant protein thioredoxin1 (Trx1). More recently and in line with this, miR-129-5p has been shown to function as a tumor suppressor when overexpressed in lung cancer cells [191]. Furthermore, chidamide, an oral benzamide-type selective HDAC inhibitor, has been shown to induce cancer cell cycle arrest by inducing upregulation of miR-129-3p together with ROS production to inhibit telomerase activity [192].

MiR-181c has been found to be deleterious in the cardiac setting, but protective in the pulmonary system. Das et al. delivered miR-181c into rats by using nanoparticles and found that it targeted the mitochondrial cytochrome c oxidase subunit 1 (mt-COX1). COX is the last enzyme of the mitochondrial respiratory chain and the major oxygen consumer enzyme in the cells [193,194]. Indeed, by delivering miR-181c, the authors observed a significantly aberrant consumption of oxygen, ROS production, and mitochondrial membrane potential in cardiac mitochondria isolated from miR-181c-nanoparticle-treated animals, suggesting that miR-181c targets mitochondrial genes, therefore causing cardiac dysfunction [195]. In the lung, miR-181c expression levels were found to be low in the tissue of COPD patients and overexpression of this miRNA was shown to inhibit cigarette smoke-induced COPD in mice. MiR-181c was found to target CNN1 (Cysr61) and its overexpression to decrease the inflammatory response, neutrophil infiltration, and inflammatory cytokines induced by cigarette smoking, as well as the reactive oxygen species (ROS) generation [196]. However, the exact mechanism by which miR-181c regulates ROS in COPD has not yet been elucidated. Of note, another member of the miR-181 family was also found to cause a reduction of the levels of ROS in the pulmonary system. Jiang et al. discovered that the expression of miR-181a was downregulated in lungs of LPS-challenged mice and that the Toll-Like Receptor 4 (TLR4) was a target of miR-181a. When miR-181a was overexpressed through a mimic transfection, the LPS-induced inflammatory response was alleviated. The authors found that overexpression of miR-181a reduced the LPS-induced intracellular ROS accumulation, similarly to what happened by siTLR4 transfection. Finally, this study suggested that miR-181a could reduce LPS-induced inflammation by targeting TLR4 and subsequently reduce ROS accumulation [197].

Finally, while the expression of miR-31-5p in the heart has been found to be induced through hypoxia and oxidative stress, a protective function for miR-31-3p was reported in the lung. In the heart, miR-31-5p has been shown to target cardiac troponin-T (Tnnt2), mineralcorticoid receptor (Nr3c2), E2F transcription factor 6 (E2f6), and metalloproteinase inhibitor 4 (Timp4). The silencing of miR-31 after myocardial infarction has been found to be beneficial and to ameliorate the left ventricle dysfunction [198]. In contrast, the silencing of miR-31-3p was reported to induce ROS production in lung cells treated with LPS. Guo et al. treated MRC-5 lung fibroblasts with LPS showing that LPS can induce cell death, cytokines expression, and ROS production in lung fibroblasts. Interestingly, they found that this phenotype was due to the overexpression of a circular RNA, circANKRD36, that acted as a sponge for miR-31-3p. CircRNAs are noncoding RNAs that bind to miRNAs to inhibit their binding to the targets. MiR-31-3p was found to target MyD88, therefore downregulating the NF-κB pathway. Thus, apoptosis and ROS production observed in LPS-treated MRC5 were caused, at least in part, by the circANKRD36 binding with miR-31-3p that impeded the miRNA downregulation of the NF-κB pathway [199].

### 4.1. MiR-155 and Oxidative Stress in Cardiac and Pulmonary Diseases

The extensive role of miR-155 in cancer (e.g., lung cancer), infections, nervous system disorders, immune system-associated diseases, and cardiovascular conditions has been recently reviewed by Gulei et al. [200]. MiR-155 was initially identified as a B-cell integration cluster (bic) capable of inducing leukosis in chickens under viral infection [201], and was subsequently found expressed in humans and mice. In the immune system, miR-155 has been shown to travel between immune cells via exosomes contributing to the regulation of inflammation [202], while in cancer it has been identified as a biomarker and associated with drug resistance [203]. Hence, numerous in vitro and in vivo studies focused on the modulation of miR-155 expression and preclinical results indicate that inhibition of miR-155, either in combination with other therapeutic approaches or by itself, might indeed be beneficial in several pathological conditions [200].

As previously mentioned, transcription factors of the FoxO family are involved in the regulation of cellular stress responses and promotion of the antioxidant defense. Herein, we underline the negative regulation of FoxO transcription factors by miR-155 in both lung and cardiac pathologies. In cardiomyocytes, miR-155 was found to directly target FoxO3a [204], ultimately leading to cardiac hypertrophy. Resveratrol (Rsv), a natural phenol, could counteract this process by directly acting on cardiomyocytes or indirectly by reducing pulmonary hypertension [205]. In line with this, Fan et al. demonstrated that miR-155 was decreased in cardiomyocytes treated with Rsv, suggesting that the beneficial anti-hypertrophic effect of Rsv is mediated by the miR-155 downregulation together with the enhanced expression of BRCA1, a key regulator of cardiac function [206], also known to be involved in lung cancer [207]. Similarly, miR-155 overexpression has been shown to inhibit FoxO1 in NSCLC [208]. Likun et al. demonstrated that FoxO1 is a direct target of miR-155, and that a miR-155/FoxO1/ROS axis exists and that it promotes NSCLC growth. In fact, antioxidant NAC treatment induced miR-155 expression, targeting FoxO1 and significantly reducing cell proliferation, therefore suggesting that the miR-155/FoxO1/ROS axis might be a novel therapeutic target for the inhibition of NSCLC growth.

Besides silencing genes from the FoxO family, miR-155 is a major player in inflammatory events, as introduced above. In an inflammatory-related context during I/R injury, increased levels of miR-155 were found to lead to the downregulation of its direct target, the suppressor of cytokines signaling 1 (SOCS-1), whose aberrant regulation contributed to the progression from hypertrophy to heart failure [209]. Using a mouse model, in vivo depletion of miR-155 caused a reduced necrosis after induction of MI and a decreased inflammatory response to injury. Interestingly, a reduced generation of ROS was measured in immune cells in this mouse model as well. The authors concluded that miR-155 could increase the inflammatory response during tissue damage by targeting SOCS-1 and, finally, by regulating the generation of ROS [210]. In line with its role in cardiac inflammation, miR-155 could play a prominent role in asthma. Indeed, elevated levels of miR-155 have also been reported in the serum of asthmatic patients [211], and it has been suggested that miR-155 modulates cockroach allergen and oxidative stress-induced COX-2 in asthma. However, although the authors showed that cockroach extract (CRE) could induce ROS production through overexpression of COX-2 and that increased ROS levels were significantly reduced in miR-155 knock out (KO) mice, it remains unclear how miR-155 regulates COX-2. In fact, they also proved direct targeting of COX-2 by miR-155 using luciferase assay and miRNA-mRNA pull-down assays and observed a lower recruitment of inflammatory cells in the lungs of CRE-treated miR-155 KO mice. They concluded that miR-155 may act to exacerbate Th2-associated lung inflammation by inducing the upregulation of its target COX-2. However, while in some cases mRNA overexpression of a miRNA target can happen to compensate for the protein downregulation, to the best of our knowledge there is no evidence for protein overexpression as a result of miRNA binding to the target 3’UTR. A better explanation for the protein overexpression of a miRNA target is that in some specific contexts the miRNA does not target the gene. In fact, each miRNA is capable of recognizing target sequences on different genes and based on cellular and molecular conditions it may or may not bind to the target. Although it is controversial whether COX-2 direct targeting underlies the correlation between miR-155 and oxidative stress during CRE exposure, overexpression of miR-155 is associated with oxidative stress in allergen-induced lung inflammation, making miR-155 inhibition a therapeutic target for the treatment of asthma.

### 4.2. MiR-21 and Oxidative Stress in Cardiac and Pulmonary Diseases

MiR-21 is a highly expressed miRNA in mammalian cells, being associated with different types of cancer, and several studies have reported a major contribution of miR-21 to apoptosis in both heart and lung tissues in oxidative stress.

MiR-21 has been shown to play a role in oxidative stress associated with DCM. Gao et al. showed that the expression of LAZ3 (also known as BCL6), an oncogene capable of inhibiting ROS production and apoptosis during chemotherapy treatments [212], decreased in in vitro and in vivo models of DCM, i.e., cardiomyocytes stimulated with HG and the heart of a DCM mouse model [213], respectively. Furthermore, the authors proved that silencing of LAZ3 in vitro led to decreased cell viability due to increased apoptosis. Specifically, the lack of LAZ3 increased oxidative stress by upregulation of NOX expression, by MDA production and by decreased activity of SOD. In line with this, Gao et al. also showed that overexpression of LAZ3 in cells or in the heart of diabetic mice attenuated oxidative stress and cell death. The mechanism leading to the LAZ3-dependent oxidative stress involved miR-21, which was shown to be decreased by LAZ3, resulting in PPARα activation, which in turn increased PGC-1α and subsequent Nrf2 expression and nuclear translocation [213]. Altogether, the authors elegantly demonstrated that miR-21 acts upstream of the PPARα/PGC-1α/Nrf2 to prevent the activation of the Nrf2 antioxidant program, leading to a deteriorating phenotype. LAZ3 inhibitory effect on miR-21 counteracts DCM progression, suggesting a potential therapeutic role of the oncogene in DCM treatment.

In contrast with a pro-oxidative role for miR-21, another study reported its protective function in the heart. Gelsolin, an actin-binding protein, inhibits the enzymatic activity of Cu/Zn SOD resulting in the accumulation of oxygen free radicals [214], and its deficiency can improve cardiac systolic function [215]. By directly targeting Gelsolin, miR-21 was able to reduce ROS production and upregulate the levels of NO available. Moreover, miR-21 alleviated palmitate-induced injury, protecting against cardiac hypertrophy in diabetic mice exhibiting diastolic dysfunction [216].

In line with this, in pulmonary vascular smooth muscle cells (VSMC) undergoing oxidative stress, miR-21 has been reported to target PDCD4, exerting a protective role as it does in cardiac myocytes [217] and human aortic endothelial cells (HAEC) [218]. In the lung, chronic hypoxia causes a massive ROS production leading to pulmonary oxidative stress, which results in pulmonary vascular remodeling [219,220]. Sarkar et al. found that hypoxia could induce the proliferation of pulmonary arterial smooth muscle cells (PASMC) through the upregulation of miR-21 [221]. Therefore, miR-21 was upregulated and actively participated in ROS response during pulmonary remodeling [220].

As reported above, it is well established that cancer cells evade cell death by taking advantage of ROS-mediated signaling pathways that allow for cancer progression by promoting proliferation [222]. In contrast with its protective role in VSMCs, miR-21 was also found to play a pro-oxidative function in cancer. Three independent studies on lung cancer have demonstrated that ROS-dependent upregulation of miR-21 is associated with poor prognosis [223,224,225] and that it occurs through the AKT [226], ERK/NF-kB [227] and MAPK [228] pathways. Zhang et al. [223] showed that a TLR4/ROS/miR-21 pathway promoted tumor progression in human lung cancer cells treated with LPS. Pulmonary infections of Gram-negative bacteria are common in lung cancer patients and elevated ROS production is critical for LPS to be able to induce miR-21 expression, promoting primary lung cancer outgrowth through miR-21 targeting of PTEN and PDCD4 [223]. Poyil et al. [224] reported that lung cells exposed to hexavalent chromium compounds Cr(VI), widely used in industry (e.g., plating, paint, steel, tanning and chrome ore processing) and classified as human carcinogens, exhibited increased levels of miR-21 expression with a consequent inhibition of PDCD4 and malignant cell transformation. Interestingly, antioxidants such as CAT were able to inhibit chronic Cr(VI)-induced miR-21 elevation and PDCD4 suppression, demonstrating that ROS have a crucial role in the regulation of the miR-21/PDCD4 signaling in lung cancer, which the authors suggested to happen through IL-6/STAT3 [224]. In a follow-up study, Poyil et al. [225] further demonstrated that quercetin, the most abundant plant food flavonoid, inhibited the Cr(VI)-induced miR-21/PDCD4 regulation by inhibiting the Cr(VI)-induced ROS generation as well [225].

Another aspect of lung cancer that has been associated with the ROS/miR-21 regulation is relative to the radiotherapy for cancer treatment. The radiation-induced bystander effects (RIBEs) is a phenomenon that may interest normal and/or cancer cells. Understanding RIBEs regulation may have important clinical implications in the context of radiotherapy. TGF-β1 cytokine has been identified as a soluble signaling molecule that mediates the RIBEs between the signaling and bystander cells [229,230,231,232] leading to increased ROS levels in the bystander cell [233]. Jiang et al. found that mir-21 mediated this signaling by being expressed in the bystander cells in response to the TGF-β1 pathways activated in both the signaling and the bystander cells, leading in turn to increased ROS levels and DNA damage. Interestingly, the authors found that the time at which the radiation conditioned media (RCM) from the signaling cells was harvested post-irradiation determined the effect on the expression of miR-21 in the bystander cells. Short-term RCM induced elevated miR-21, and ROS levels and elevated DNA damage in the bystander cells, while miR-21 levels were reduced and proliferation inhibited in bystander cells following exposure to the long-term RCM [233]. Thus, timing and regulation of miR-21 expression, from which subsequently the oxidative stress in bystander cells depends, might be crucial in radiotherapy. Finally, miR-21 has also been reported to target SOD3 and TNF-α [234] to modulate the levels of ROS, suggesting that a complex crosstalk between miR-21 and ROS is likely to exist.

### 4.3. MiR-1/206 and Oxidative Stress in the Cardiac and Pulmonary Diseases

MiR-1 and miR-206 are evolutionarily conserved miRNAs exhibiting a high sequence and expression similarity in the muscle from *C. elegans* to human, even if they are not located on the same genomic region. Although these two miRNAs originate from different pre-miRNA sequences, their mature sequences vary in only four nucleotides, exhibiting identical seed sequence and thus sharing many target genes. However, their role is controversial since they can act either separately, being implicated in multiple regulatory pathways, or together to regulate the same genes [235].

Both miR-1 and miR-206 have a prominent role in the regulation of the antioxidant system. Wang et al. [236] were interested in studying whether miR-1 levels could affect proteins related to oxidative stress and directly influence heart dysfunction. In miR1-overexpressing mice, ROS levels were elevated and the activity of enzymes such as LDH and CK in plasma were found to be increased. Accordingly, the authors also found that inhibiting miR-1 levels in rat cardiomyocytes could revert these results. On the contrary, overexpression of miR-1 on such cells led to increased ROS levels and apoptosis, following treatment with H_2_O_2_. The authors also found that the redox-related proteins, SOD1, glutamate-cysteine ligase catalytic subunit (Gclc), and glucose-6-phosphate dehydrogenase (G6PD) were targets of miR-1. In the lung, SOD1 is also targeted by miR-206 that shares the same locus with miR-1. Specifically, Wanga et al. demonstrated that miR-206 expression levels are increased upon ROS production induced by PM2.5 in an asthmatic mouse model. They identified SOD1 as the genuine target of mir-206, suggesting that the PM2.5/miR-206/SOD1 regulation axis causes ROS accumulation and disease progression in asthmatic mice [237].

Metabolism homeostasis is critical in cardiac and lung tissue. For instance, cardiac metabolism includes various biochemical reactions necessary to sustain cardiomyocyte growth or contraction, while the lung, besides its function of gas exchange machinery, can metabolize vasoactive molecules (i.e., angiotensin). Therefore, a fine-tuning of metabolic signaling pathways is paramount for maintaining a proper cardiac and pulmonary function. Here, we summarize some elegant studies that shed light on the critical impact of miR-1 and miR-206 on cardiac or pulmonary metabolism. High-fat diet or Liver X Receptors (LXRs) are key regulators of lipid and glucose metabolism and also have an anti-inflammatory role. Activation of LXRs in the heart attenuates cardiovascular diseases, such as DCM in diabetic mice [238]. A synthetic agonist of LXRα has been shown to attenuate oxidative stress, mitochondrial damage, and apoptosis in cardiomyocytes in HG conditions. What adds to the interest is that in glucose-induced cells, miR-1 has been found to be overexpressed and to target LXRα, thus enhancing apoptosis and ROS production; it increased mitochondrial membrane potential and aggravated the cleavage of PARP, caspase-3, and caspase-9 [97]. All such effects could be prevented by IGF-1, which has been shown to be itself a direct target of miR-1 [239]. Within the pulmonary system, gain of function of Nrf2 has been shown to promote tumorigenesis. Singh et al. showed that, in response to metabolic stress, lung cancer cells activated Nrf2 signaling through an autoregulatory feedback loop involving miRNA-dependent regulation of genes involved in glucose metabolism pathways, i.e., the pentose phosphate pathway (PPP), the tricarboxylic acid (TCA) cycle, and fatty acid synthesis. Prolonged Nrf2 activation reduced downstream miR-1 and miR-206, that in turn enhanced the expression of metabolic genes such as G6PD, transketolase (TKT), phosphogluconate dehydrogenase (PGD), and glycerol-3-phosphate dehydrogenase (GPD2) in both malignant and nonmalignant cells [240].

Finally, the amount of ROS is a determining factor to modulate expression of miRNAs and their target genes that are important effectors during pathogenesis. During cardiac hypertrophy or I/R, Lee et al. [241] showed that varying the intensity and the frequency of ROS dosages resulted in differential expressions of miR-1 and in decreasing levels of its target, myocardin. In vivo, miR-1 overexpression attenuated TAC-induced cardiac hypertrophy, while its inhibition attenuated I/R-induced cardiac apoptosis.

## 5. Conclusions

The present review extensively summarizes the importance of the crosstalk between miRNAs and ROS in cardiac and pulmonary diseases. Interestingly, in both cardiac and pulmonary settings, miRNAs have been shown to interfere with the generation of ROS and oxidative stress using a dual mechanism of action: (1) directly, by targeting oxidase and antioxidant enzymes (e.g., NOX, SOD, CAT), antioxidant genes (e.g., Sirt, Trx1) and their transcription factors (e.g., Nrf2, FOXOs), as well as mitochondrial genes (e.g., COX, Bnip3), and (2) indirectly, mainly by targeting genes involved in apoptosis (e.g., Bcl2, NFκB), tumor–suppressor genes (e.g., p53), and interfering with pro-survival signaling pathways (e.g., Akt, IGF-1). Furthermore, miRNA expression itself can be either induced directly by ROS or modulated in response to inflammatory pathways and contribute ultimately to oxidative stress. It is also worth considering that, among the miRNAs linked to oxidative stress in the pulmonary system, there are some known to play a role in cardiac diseases; however, not yet correlated with oxidative stress. The same is true for some miRNAs involved in pulmonary diseases that were found to be correlated to oxidative stress in the cardiac settings. Finally, among the miRNAs playing a role in oxidative stress in both systems, miR-421, miR-129, and miR-181c were found to have an opposite role in cardiac vs. pulmonary diseases, supporting the existence of a complex crosstalk between miRNAs and ROS that may depend on tissue specificity.
